# Development of a Machine Learning Model Using Multiple, Heterogeneous Data Sources to Estimate Weekly US Suicide Fatalities

**DOI:** 10.1001/jamanetworkopen.2020.30932

**Published:** 2020-12-23

**Authors:** Daejin Choi, Steven A. Sumner, Kristin M. Holland, John Draper, Sean Murphy, Daniel A. Bowen, Marissa Zwald, Jing Wang, Royal Law, Jordan Taylor, Chaitanya Konjeti, Munmun De Choudhury

**Affiliations:** 1 Department of Computer Science and Engineering, Incheon National University, Incheon, South Korea; 2 Office of Strategy and Innovation, National Center for Injury Prevention and Control, Centers for Disease Control and Prevention, Atlanta, Georgia; 3 Division of Violence Prevention, National Center for Injury Prevention and Control, Centers for Disease Control and Prevention, Atlanta, Georgia; 4 National Suicide Prevention Lifeline, New York, New York; 5 National Center for Environmental Health, Centers for Disease Control and Prevention, Atlanta, Georgia; 6 School of Interactive Computing, Georgia Institute of Technology, Atlanta

## Abstract

**Importance:**

Suicide is a leading cause of death in the US. However, official national statistics on suicide rates are delayed by 1 to 2 years, hampering evidence-based public health planning and decision-making.

**Objective:**

To estimate weekly suicide fatalities in the US in near real time.

**Design, Setting, and Participants:**

This cross-sectional national study used a machine learning pipeline to combine signals from several streams of real-time information to estimate weekly suicide fatalities in the US in near real time. This 2-phase approach first fits optimal machine learning models to each individual data stream and subsequently combines predictions made from each data stream via an artificial neural network. National-level US administrative data on suicide deaths, health services, and economic, meteorological, and online data were variously obtained from 2014 to 2017. Data were analyzed from January 1, 2014, to December 31, 2017.

**Exposures:**

Longitudinal data on suicide-related exposures were obtained from multiple, heterogeneous streams: emergency department visits for suicide ideation and attempts collected via the National Syndromic Surveillance Program (2015-2017); calls to the National Suicide Prevention Lifeline (2014-2017); calls to US poison control centers for intentional self-harm (2014-2017); consumer price index and seasonality-adjusted unemployment rate, hourly earnings, home price index, and 3-month and 10-year yield curves from the Federal Reserve Economic Data (2014-2017); weekly daylight hours (2014-2017); Google and YouTube search trends related to suicide (2014-2017); and public posts on suicide on Reddit (2 314 533 posts), Twitter (9 327 472 tweets; 2015-2017), and Tumblr (1 670 378 posts; 2014-2017).

**Main Outcomes and Measures:**

Weekly estimates of suicide fatalities in the US were obtained through a machine learning pipeline that integrated the above data sources. Estimates were compared statistically with actual fatalities recorded by the National Vital Statistics System.

**Results:**

Combining information from multiple data streams, the machine learning method yielded estimates of weekly suicide deaths with high correlation to actual counts and trends (Pearson correlation, 0.811; *P* < .001), while estimating annual suicide rates with low error (0.55%).

**Conclusions and Relevance:**

The proposed ensemble machine learning framework reduces the error for annual suicide rate estimation to less than one-tenth of that of current forecasting approaches that use only historical information on suicide deaths. These findings establish a novel approach for tracking suicide fatalities in near real time and provide the potential for an effective public health response such as supporting budgetary decisions or deploying interventions.

## Key Points

**Question:**

Can real-time streams of secondary information related to suicide be used to accurately estimate suicide fatalities in the US in real time?

**Findings:**

In this national cross-sectional study, combining information from 8 data streams encompassing various health services and online data sources enabled accurate, real-time estimation of US suicide fatalities with meaningful correlation to week-to-week epidemiological trends and a less than 1% error compared with actual counts.

**Meaning:**

These findings advance the first efforts to create a population-level system for enabling real-time epidemiological trend monitoring of suicide fatalities.

## Teaser

This cross-sectional study uses machine learning to estimate weekly suicide fatalities in the US in near real time.

## Introduction

Suicide is a significant contributor to global mortality, resulting in nearly 800 000 deaths worldwide each year.^1^ Currently, suicide rates in the US are at their highest levels in more than 50 years after having experienced a relatively rapid, recent increase of 35% from 1999 to 2018 alone.^2,3^ Despite the urgency of this public health problem, real-time information on suicide fatality trends to guide prevention efforts is lacking. This is because national statistics on suicide rates are delayed by 1 to 2 years.^4,5^ Official statistics on suicide fatalities are produced by the Centers for Disease Control and Prevention using data from the National Vital Statistics System. The National Vital Statistics System collects information from death certificates that are submitted by states to the Centers for Disease Control and Prevention from more than 2000 medical examiner and coroner offices in the US.^6^ Reasons for the delay in suicide fatality data include the decentralized nature of mortality statistics, critical shortages of workers such as forensic pathologists, variation in local information technology infrastructure, and the time needed to investigate deaths due to suicide.^4,7,8^

Lagged mortality data presents several challenges to public health. First, it prevents federal agencies from making budgetary decisions that are accurately matched to the current magnitude of the problem. In addition, without the ability to detect rapid changes in mortality trends, a timely and coordinated national-level response to support and implement prevention programs and policies, such as those that strengthen economic supports, increase access to care and interventions, enhance social connectedness, and improve identification of those at risk, among other strategies described in Centers for Disease Control and Prevention’s technical package for suicide prevention,^9^ are not possible. Thus, there exists a significant need to develop and test the feasibility, accuracy, and applicability of novel methods that can provide a more real-time understanding of suicide trends to inform public health activities and budgetary decision-making.

To date, limited work has evaluated and validated approaches to produce more real-time estimates of mental illness or suicide. Owing to the social and environmental underpinnings of suicide,^10^ some of the earliest efforts to examine real-time trends related to suicide focused on using signals from large-scale social media data, such as Twitter.^11,12^ In recent years, social media data have been backed by a rich literature demonstrating its potential in assessing and inferring a wide variety of mental health attributes and outcomes.^13,14,15,16,17,18,19,20,21,22,23,24^ Notwithstanding this evidence, the potential of social media data in supporting public health efforts for population-level suicide assessment has yet to be fully harnessed. Moreover, methods that use social media data alone can be limited by issues of proxy and sampling bias,^25^ a lack of demographic and geographic representativeness with respect to the general population,^26^ structural idiosyncrasies owing to different platforms’ distinct affordances,^27^ and epistemological issues around what social media–derived signals really mean when taken out of context.^28^

Other online data sources, such as Google search trends, have also been evaluated for use in tracking suicide trends, and findings have revealed mixed results depending on the method used; studies have generally only used cross-sectional approaches and have not tested such data for prospective prediction tasks.^29,30,31^ Furthermore, public health use of Google trends as a stand-alone data source has come under criticism in recent years, following unanticipated challenges in estimating prevalence of influenzalike illness.^32^

Consequently, researchers have suggested that greater value can be obtained by combining online data with other near–real-time health data to facilitate better public health monitoring.^32,33^ To this end, researchers have attempted to strengthen and supplement the signal produced from online search or social media data by examining variables that provide additional population-level environmental context for suicide risk, such as macroeconomic indicators and meteorological patterns.^11,34^ To our knowledge, no study has attempted to combine information from disparate real-time data sources to evaluate the ability of multiple streams to tackle the pressing public health need of enabling estimation of suicide fatality rates for the US in near real time.

In this study, we aim to evaluate the individual and combined ability of several disparate categories of real-time health services and economic, meteorological, and online data sources to produce weekly estimates of the number of suicide fatalities in the US. Such an ensemble approach will likely allow countering the underlying biases (eg, sampling bias or demographic/geographic misrepresentation idiosyncrasies) of each of the individual data sources, because suicidal outcomes are multifaceted and subpopulations are likely to be represented and stratified differentially in each data source.

## Methods

### Data Sources

For this cross-sectional study, we started with a comprehensive set of data sources spanning the categories above, which were selected in a theoretical and domain-inspired way and from prior literature.^35,36,37,38^ Because these were public and/or deidentified data, the study did not constitute human subjects research, and therefore the ethical review board of Georgia Institute of Technology considered the research exempt. This cross-sectional study followed the Strengthening the Reporting of Observational Studies in Epidemiology (STROBE) reporting guideline.

First, our work harnessed an underexplored class of real-time data that can provide insight into suicide epidemiology: administrative data generated from the provision of clinical or behavioral support services.^39^ The 3 health services data sources we used were (1) weekly proportion of emergency department visits for suicide ideation or attempt as documented in electronic health record data from the National Syndromic Surveillance Program (2015-2017)^40^; (2) weekly volume of calls made to the National Suicide Prevention Lifeline, a national telephone hotline for mental health crises (2014-2017)^41^; and (3) weekly proportion of calls to US poison control centers as captured in the National Poison Data System for an exposure attributed to intentional self-harm of all poison control center exposure calls (2014-2017).^42^ Although these data sources have been used to support traditional epidemiological studies, such data have thus far not been evaluated for suicide prediction tasks.

Second, for economic data, we considered several indicators available with monthly frequency from 2014 to 2017 through Federal Reserve Economic Data, including consumer price index and seasonality-adjusted unemployment rate, overall hourly earnings, hourly earnings in manufacturing, home price index, and 3-month and 10-year yield curves.^43^ Third, we assessed a key meteorological indicator, the duration of daylight hours (mean number for each week; 2014-2017) per prior work.^11,44^ These data sources are not discussed in detail herein because they either lack temporal immediacy to be actionable in weekly estimation of fatalities or do not capture variability well in the magnitude of fatalities; therefore, they were found to not contribute meaningfully to improved model performance. Details are given in eMethods 1 and 3 in the Supplement.

Fourth, we used online data, which included Google trends data (weekly normalized popularity scores of searches spanning 42 suicide-related terms on the Google search engine; 2014-2017), YouTube trends data (weekly normalized popularity scores of searches spanning 42 suicide-related terms on YouTube; 2014-2017), public Twitter data (weekly number of Twitter posts containing any of 38 suicide-related keywords, phrases, and hashtags [9 327 472 tweets]; 2015-2017;), public Reddit data (weekly normalized fraction of posts shared on 55 suicide- and mental health–related subcommunities on Reddit/subreddits [2 314 533 posts]; 2014-2017), and public Tumblr data (weekly normalized fraction of posts related to 42 suicide-related key words, phrases, and hashtags [1 670 378 posts]; 2014-2017). To normalize the suicide-related data corresponding to each of these sources, the Google and YouTube trends data were restricted to queries made in the US; however, for Reddit, Twitter, and Tumblr, we did not impose this constraint, because country-specific use statistics for these platforms are not available. We do not consider this to be a significant issue, because most of the users in these social media platforms are from the US. Detailed information on the acquisition and processing of each data source, including their descriptive statistics, can be found in eMethods 1 and eTables 1-3 in the Supplement. The time-series values of the individual data sets that are included and excluded in the proposed model are plotted in eFigures 3 and 4 in the Supplement, respectively.

Our primary outcome of interest was weekly counts of suicide fatalities in the US. These data were available from 2010 through 2017. Suicide deaths were identified from death certificate data from the National Vital Statistics System using the *International Statistical Classification of Diseases and Related Health Problems, Tenth Revision*, underlying cause of death codes U03, X60-X84, and Y87.0.

### Machine Learning Approach

**Figure 1.**
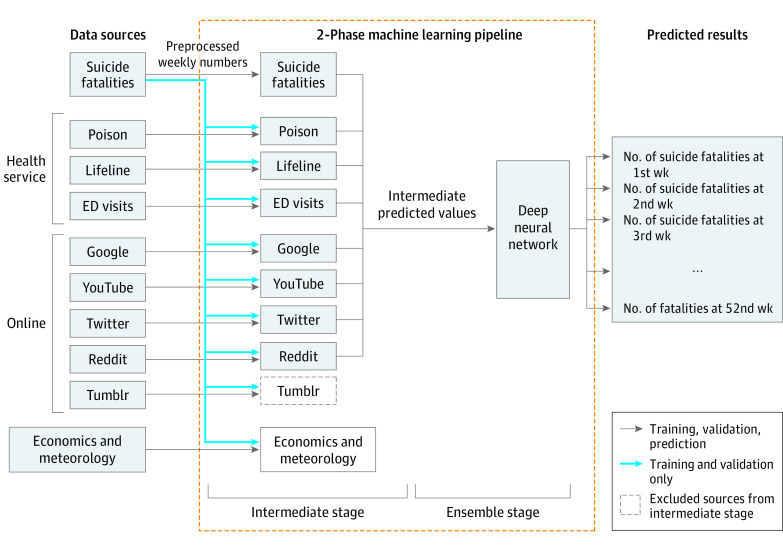
Overall Architecture of Machine Learning Pipeline Framework to Estimate Weekly Suicide Fatalities In the first or intermediate phase, we identified the best model corresponding to each data source by training and validating a number of state-of-the-art machine learning models for each data source. The predicted estimates of weekly suicide fatalities given by a single data source were then combined in an automatic and harmonic way via a neural network model in the ensemble (second) phase. Health services include the National Suicide Prevention Lifeline (Lifeline), US poison control center calls (Poison), and emergency department (ED) visits.

We developed a novel 2-phase machine learning pipeline to leverage these data sets for near–real-time estimation of weekly suicide fatalities (Figure 1). In the first phase, we developed the best model corresponding to each data source. We set aside weekly suicide fatalities data from 2017 as our held-out test data for evaluating final performance, 2016 fatalities data for validation, and data from 2015 and before (if available) for training the prediction models. Predictive features for training, validation, and testing for each of the clinical and online data sources were constructed using a lagged (value at a previous time step or previous week) sliding-window approach,^45^ wherein we used weekly data from the various data sources as features for the modeling/estimation task of suicide fatalities in the same week, because these data sets can be gathered in near real time. In addition, we leveraged historical data on weekly suicide fatalities as an additional data source for our estimation task, both to augment the estimates given by the real-time signals as well as to develop a baseline model for comparison. We trained and validated a number of leading machine learning models: linear regression, LASSO (least absolute shrinkage and selection operator), ridge, elastic net,^46^ random forest regression, and support vector regression.^47^ In the second phase, using an ensemble machine learning modeling approach, we combined the model outputs (the best predicted estimates of weekly suicide fatalities given by a single data source) from the first phase in an automatic and harmonic way via a neural network model to obtain the final estimates. The rationale of this approach is in line with super learner algorithm,^48^ which finds the best combination among different models. eMethods 2, eFigure 1, and eTable 4 in the Supplement provide detailed information of this machine learning model development approach.

## Results

Table 1 shows the performance of the individual models on the held-out 2017 test data at the conclusion of the first stage (see eMethods 3 and eTable 6 in the Supplement for extended results). We do not include performance of the models built using the economic and meteorological data sets herein, because in the ensemble step, these data sources degraded performance; therefore, we excluded them in the subsequent discussion (see eMethods 3 and eTable 5 in the Supplement).

**Table 1.** Performance of Individual Models Built Using Each Data Source at the Intermediate Stage (First Phase)

Category	Source	Pearson correlation coefficient	RMSE	MAPE, %	SMAPE, %	Annual estimated rate per 100 000 people (error, %)
Historical suicide fatalities	NA	0.761	79.790	7.485	3.941	13.35 (7.80)
Health services data	Poison control center calls	0.702	162.019	17.103	9.409	11.95 (17.47)
	National Suicide Prevention Lifeline	0.491	83.871	7.256	3.419	15.27 (5.46)
	ED visits	0.511	54.353	4.894	2.441	14.38 (0.69)
Online data	Google (all categories)	0.721	85.040	7.846	4.135	13.28 (8.29)
	YouTube (mental health)	0.580	155.892	13.683	7.715	12.65 (12.64)
	Reddit	0.507	210.986	22.255	12.596	11.19 (22.72)
	Twitter	0.389	72.640	6.466	3.355	13.75 (5.04)

Abbreviations: ED, emergency department; MAPE, mean absolute percentage error; NA, not applicable; RMSE, root mean square error; SMAPE, symmetric mean absolute percentage error.

In addition, as a baseline, we also report the results from the best model trained using only historical suicide fatality data (as baseline models, we considered linear regression, support vector regressor, and Holt-Winters models to factor in seasonality). Although the baseline model has the highest Pearson correlation coefficient (0.761; *P* < .001) of all individual models owing to the strong seasonality of suicide (the maximum difference of correlation coefficient is 0.372), such a model built using only lagged historical data cannot detect real-time changes in suicide rates and significantly underestimated the growth in suicide rates in 2017.

Examining the other data sources, we found each individual data set to have distinct strengths and weaknesses (eTable 7 in the Supplement) in (1) tracking seasonality (week-to-week) trends as measured by the Pearson correlation coefficient; (2) minimizing the error of weekly estimates (as measured by root mean squared error [RMSE], mean absolute percentage error [MAPE], and symmetric MAPE [SMAPE]); and (3) estimating the total number of suicide fatalities during an entire year as measured by the percentage error in the annual estimated rate of suicide per 100 000 people. For example, the Pearson correlation coefficients for predictions made from both the poison and Google data with actual weekly suicide fatality counts were high (0.702 and 0.721, respectively; *P* < .001 for both); however, RMSE and MAPE for these data sources are higher than some other sources (the maximum differences of RMSE and MAPE are between poison control center calls and emergency department visits, which are 107.666 and 12.209, respectively), indicating higher variance week to week. Conversely, although the emergency department visit data show a slightly more modest Pearson correlation coefficient (0.511; *P* < .001), mean weekly percentage error estimates are low (MAPE, 4.894%). These results show that each individual data source can contribute unique and complementary advantages to a model estimating suicide fatalities.

Table 2 shows ensemble models combining predictions via a neural network model. As expected, all of the ensembles improve over models that use individual data sources alone; the Pearson correlation coefficient rises to 0.811 (*P* < .001) for the all data sources ensemble with a corresponding error of only 0.55% in estimating the annual rate of suicide fatalities, from a maximum Pearson correlation coefficient of 0.761 (and a corresponding error of 7.80%) in the case of the individual models. The all data sources ensemble outperforms predictions made from the best performing baseline model (increase by 0.05 in terms of correlation coefficient and decrease by 35.351 and 7.25 in terms of RMSE and error percentage of annual estimated rate, respectively; *P* < .001), which makes estimates from historical suicide fatality data. Specifically, the all data sources ensemble model improves the correlation while also reducing error around weekly estimates by approximately half and the error for the annual estimate to less than one-tenth of that of the baseline model.

**Table 2.** Performance of the 6 Ensemble Models Built Using Different Combinations of the Data Sources

Ensemble type^a^	Pearson correlation coefficient	RMSE	MAPE, %	SMAPE, %	Annual estimated rate per 100 000 people (error, %)
Health services data sources	0.802	56.847	5.457	2.629	15.08 (4.14)
Online data sources	0.633	52.035	4.189	2.149	14.12 (2.49)
Baseline plus health services sources	0.832	46.096	4.367	2.123	14.91 (2.97)
Baseline plus online data sources	0.737	80.478	7.306	3.841	13.37 (7.67)
Health services plus online data sources	0.791	43.239	3.806	1.916	14.31 (1.17)
All data sources	0.811	44.439	4.006	2.001	14.40 (0.55)

Abbreviations: MAPE, mean absolute percentage error; RMSE, root mean square error; SMAPE, symmetric mean absolute percentage error.

^a^ Health services data sources include emergency department visits, National Suicide Prevention Lifeline, and poison control center call data. Online data sources include Google, YouTube, Reddit, and Twitter.

It is also important to note that although the ensemble of the health services data sources outperforms the online data sources ensemble in terms of correlation coefficient, the ensembled online data has a relatively low percentage error when estimating suicide fatalities. The Pearson correlation coefficient and percentage error of annual suicide rate of health services data sources are 0.802 and 4.14% (*P* < .001), respectively; those of online data sources are 0.633 and 2.49% (*P* < .001), respectively. The ensemble of combining these sources helps to reduce variance of the all data sources ensemble (difference of 3.59% of error rate from health services data sources).

**Figure 2.**
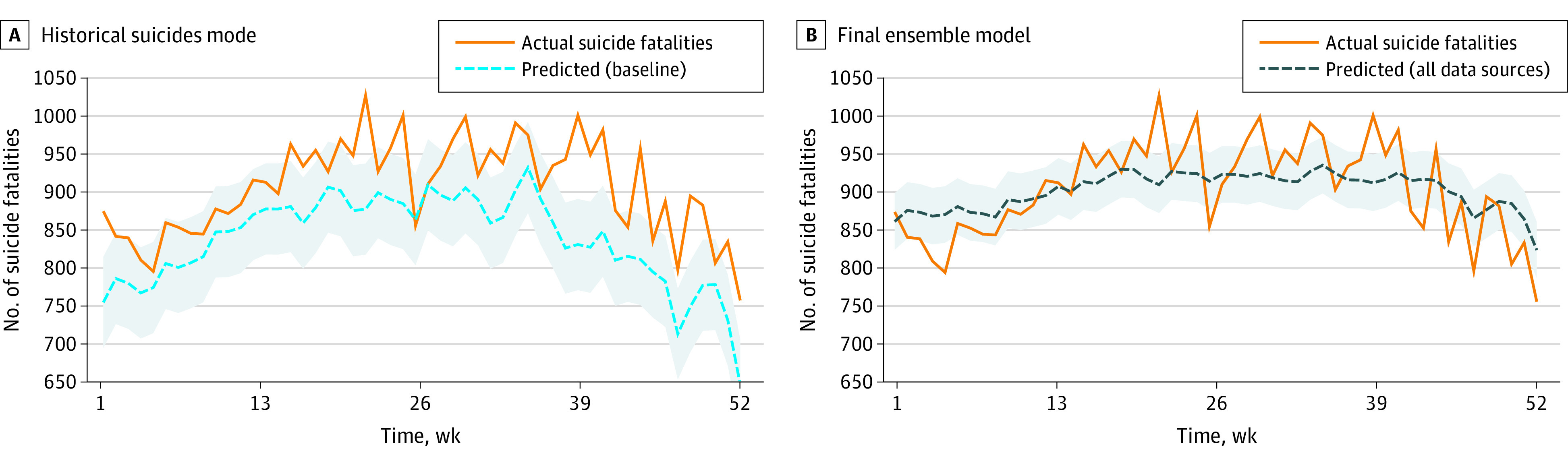
Estimation Results Weekly number of suicide fatalities estimated by the best performing historical suicide fatalities baseline model (A), which consistently underestimates weekly suicides, and the final ensemble model that combines all data sources (B). The gray areas represent 95% CIs.

Finally, the weekly counts of predicted (or estimated) suicide fatalities are plotted alongside actual weekly suicide fatalities, as shown in Figure 2. Additional plots showing the estimated values of suicide fatalities by the other ensembles can be found in eFigure 2 in the Supplement. The baseline univariate time series model underestimated suicide counts in our test year, meaning that suicides increased at a rate greater than that observed in prior years, but this was not learned by the baseline model. Our final ensemble model that used near–real-time data, on the contrary, not only followed the trend but also gave estimated values of suicide fatalities that are much closer to the actual values.

## Discussion

### Implications

Our work bears several promising implications for public health. First, as mentioned above, there is currently no established way to gather real-time national information on suicide trends, which is essential for timely suicide prevention efforts. Lagged data often do not reflect unanticipated shifts, in particular, the rise in suicide fatalities in recent years. By combining information from both online, big-data sources and more traditional health data sources, we are able to achieve a fairly accurate estimation of suicide fatalities in a near–real-time fashion that is less prone to the underlying biases, idiosyncrasies, and unique characteristics of any single data source.

Second, although combining multiple streams of data via machine learning models to produce disease forecasts has emerged as a leading quantitative approach in the study of influenzalike illness and a limited number of infectious diseases,^49^ it has been unclear whether such approaches are translatable to noninfectious diseases such as suicide. For example, the lag between when historical data are available and when estimates are being made is often much greater for noninfectious diseases (ie, criterion-standard influenza estimates are delayed by 1-2 weeks, whereas criterion-standard estimates of suicide are delayed by 1 or more years, as noted above). Our work demonstrates a robust quantitative approach as well as elucidates novel data sources where criterion-standard historical data lag by 1 year, suggesting promise for real-time estimation of such noninfectious diseases.

Third, unlike some limited prior efforts that have attempted to combine data sets for suicide risk estimation,^11^ our framework uses ensemble modeling that allows us to go beyond simplistic time series forecasting approaches of integrating disparate data sets (eg, in linear combinations). Our machine learning framework also uses state-of-the-art machine learning methods, such as neural networks, which are able to glean those patterns (or features) embedded in the data that may otherwise be latent or not apparent in linear or polynomial regression models. Essentially, using these techniques makes our pipeline highly flexible—should new, appropriate data streams become available or existing ones cease to be useful—as well as amenable to audits and deployment.

Finally, development and testing of our suicide fatalities estimation model mirrored real-world constraints in public health monitoring and surveillance of suicide. For this reason, we chose the Pearson correlation coefficient as a metric to choose the best model at the intermediate stage, and then for reporting the eventual best models. This is because policy makers or public health stakeholders focus on projected increases and decreases in suicide fatalities for decision-making, and these trends or patterns therefore constitute more informative and actionable signals rather than metrics that optimize for overall best means (such as RMSE). Finally, to stakeholders who are nonexperts at machine learning, correlation coefficients can be more interpretable compared with metrics such as MAPE. That said, our work highlights the tradeoffs between different performance metrics in the final set of ensemble models, which may serve as helpful resources and considerations for real-world use.

### Limitations

Despite the implications described above, this work has some limitations. First, caution is needed when generalizing data sources and estimation results to other countries, to smaller geographic units, or among specific sociodemographic groups. Next, because the use patterns and norms of social media and relevant key words change over time, models from social media or web services may be less helpful to estimate suicide fatalities in years beyond 2017, and predictive signals from newer social media platforms may be more important going forward. Consequently, real-time, real-world deployment will require periodic retraining and incorporation of new and changing data sources that emerge as leading proxy signals for mental health.

## Conclusions

Our use of multiple novel data sources with a 2-stage machine learning pipeline involving use of a neural network to identify and combine features demonstrated excellent performance. Indeed, the performance improvement beyond current standard practice is considerable. The historical fatalities model, which uses only historical data to estimate future trends and which represents contemporary public health practice, would have resulted in an overall error of 7.80% if applied to estimate suicides throughout 2017, whereas the ensemble approach we present would have resulted in a 0.55% error. This rate represents a multifold improvement beyond current modeling practice. Risk factors for and drivers of suicide rates are multifactorial, which necessitates a coordinated and comprehensive public health approach. Examining suicide from the perspective of multiple data sources, each representing a unique aspect of the problem, can help inform federal support of appropriate programs and policies to prevent suicide. For example, more timely information about rapidly increasing suicide trends could enable governmental funding and support for programs to prevent suicide in a way that better keeps pace with the growing magnitude of the problem. Such efforts might include more rapidly addressing clinician shortages in mental health care; expanding crisis intervention programs, such as hotline, chat, or text services; or strengthening policies and programs that address underlying risk factors, such as economic or housing instability.^9^ This work advances the very first efforts in achieving a near real-time understanding of suicide in the United States.

## Notes

Supplement. **eMethods 1.** Data Collection, Data Description, and Preprocessing **eMethods 2.** Machine Learning Model Development **eMethods 3.** Exploration of Alternative Data Sets **eFigure 1.** Training, Validation, and Test Sets of All of the Data Sources **eFigure 2.** Weekly Number of Suicide Fatalities Estimated by the Different Ensemble Models **eFigure 3.** Time Series Values For Historical, Social Media, and Health Services Data **eFigure 4.** Time Series Values of Meteorology, Social Media Platform, and Economics Data **eTable 1.** Socioeconomic-Behavioral Data Types and Their Associated Sources **eTable 2.** Keyword Sets (or Subreddits) for Suicide-Related Data Collection for the 5 Online Data Sources **eTable 3.** Descriptive Statistics of Online Data Sets **eTable 4.** The List of the Parameters Explored During Grid Search at the First and Second Stages **eTable 5.** Estimation Results From Individual Data Sources That Were Excluded in the Final Machine Learning Pipeline **eTable 6.** Performance of Ensemble Models Combining Home Price Index (HPI) and Number of Daylight Hours With Health Services and Online Data Sources **eTable 7.** Performance of Models Built From Each Data Source at the Intermediate Stage (First Phase) **eReferences.**
