# Supplementary material for: Development of a Machine Learning Model Using Multiple, Heterogeneous Data Sources to Estimate Weekly US Suicide Fatalities
Source: JAMA Netw Open. 2020 Dec 23;3(12):e2030932. doi: 10.1001/jamanetworkopen.2020.30932 (PMC7758810; doi:10.1001/jamanetworkopen.2020.30932)
Supplement: Supplement. — eMethods 1. Data Collection, Data Description, and Preprocessing eMethods 2. Machine Learning Model Development eMethods 3. Exploration of Alternative Data Sets eFigure 1. Training, Validation, and Test Sets of All of the Data Sources eFigure 2. Weekly Number of Suicide Fatalities Estimated by the Different Ensemble Models eFigure 3. Time Series Values For Historical, Social Media, and Health Services Data eFigure 4. Time Series Values of Meteorology, Social Media Platform, and Economics Data eTable 1. Socioeconomic-Behavioral Data Types and Their Associated Sources eTable 2. Keyword Sets (or Subreddits) for Suicide-Related Data Collection for the 5 Online Data Sources eTable 3. Descriptive Statistics of Online Data Sets eTable 4. The List of the Parameters Explored During Grid Search at the First and Second Stages eTable 5. Estimation Results From Individual Data Sources That Were Excluded in the Final Machine Learning Pipeline eTable 6. Performance of Ensemble Models Combining Home Price Index (HPI) and Number of Daylight Hours With Health Services and Online Data Sources eTable 7. Performance of Models Built From Each Data Source at the Intermediate Stage (First Phase) eReferences. [file jamanetwopen-e2030932-s001.pdf]

Supplementary Online Content

Choi D, Sumner SA, Holland KM, et al. Development of a machine learning model using multiple, heterogeneous data sources to estimate weekly US suicide fatalities. *JAMA Netw Open*. 2020;3(12):e2030932. doi:10.1001/jamanetworkopen.2020.30932

eMethods 1. Data Collection, Data Description, and Preprocessing
eMethods 2. Machine Learning Model Development
eMethods 3. Exploration of Alternative Data Sets
eFigure 1. Training, Validation, and Test Sets of All of the Data Sources
eFigure 2. Weekly Number of Suicide Fatalities Estimated by the Different Ensemble Models
eFigure 3. Time Series Values For Historical, Social Media, and Health Services Data
eFigure 4. Time Series Values of Meteorology, Social Media Platform, and Economics Data
eTable 1. Socioeconomic-Behavioral Data Types and Their Associated Sources
eTable 2. Keyword Sets (or Subreddits) for Suicide-Related Data Collection for the 5 Online Data Sources
eTable 3. Descriptive Statistics of Online Data Sets
eTable 4. The List of the Parameters Explored During Grid Search at the First and Second Stages
eTable 5. Estimation Results From Individual Data Sources That Were Excluded in the Final Machine Learning Pipeline
eTable 6. Performance of Ensemble Models Combining Home Price Index (HPI) and Number of Daylight Hours With Health Services and Online Data Sources
eTable 7. Performance of Models Built From Each Data Source at the Intermediate Stage (First Phase)
eReferences.

This supplementary material has been provided by the authors to give readers additional information about their work.

©2020 Choi D et al. *JAMA Network Open*.

eMethods 1. Data Collection, Data Description, and Preprocessing

As noted in the paper, we searched and investigated the potential utility of data from diverse sources like social media, health services, economics, and meteorology in our estimation task of national suicide fatalities. Our search and investigation used theoretical, prior-literature, and domain-inspired perspectives, which are noted for each data source type described below.

The acquisition and pre-processing of the various datasets are explained as follows. The use of the health services datasets for this research was guided by appropriate data use agreements with the relevant organizations, while for the online datasets we focused solely on public data for the social media streams. All data were further temporally and geographically aggregated and stripped of any personally identifiable information; only deidentified data was used for the subsequent analysis. The research was considered exempt by the relevant Institutional Review Board, as it did not constitute human subjects research.

Datasets from Health Services

As suicidal ideation, self-directed violence, and suicide attempts are some of the strongest predictors of suicide,[1] we hypothesized the following data sources, which provide near real-time insight from various settings about the frequency of these outcomes, would be useful in developing a model that could predict weekly suicide counts.

- **ED Visits:** Data on the daily number of suicide-related Emergency Department (ED) visits was obtained from querying data from the National Syndromic Surveillance Program (NSSP). NSSP is a convenience sample of approximately 71% of ED visits nationwide and includes free-text information on the patient's chief complaint and any discharge diagnosis codes assigned to the electronic patient encounter. Validated case-definitions have been developed in collaboration with CDC and state and local health departments and are routinely used to monitor suicide-related ED visit trends .[2] To account for temporal changes in the number of ED's participating in NSSP over time, daily counts of the number of suicide-related visits were divided by daily counts of the total number of ED visits. These normalized daily rates were then averaged by week. The timeframe of this dataset was 2015-2017.

- **Lifeline:** The National Suicide Prevention Lifeline (1-800-273-8255; https://suicidepreventionlifeline.org/) is the primary telephone hotline to provide mental health crisis and counseling services nationwide. The Lifeline receives federal funding and is comprised of a network of over 160 local crisis centers which provide toll-free services 24 hours a day. Lifeline data included in our models represent the total volume of routed calls connected to a call center each week; we use this data source as a way to capture reports of suicidal ideation and attempts. The timeframe of this dataset was 2014-2017.

- **Poison:** The National Poison Data System (NPDS) collects data from all calls to each of the nation's 55 poison control centers. Poison control centers are telephone helplines that both citizens or health professionals may call

to seek guidance after a substance exposure. This service is free of charge. As part of routine data collection, poison control centers record whether the exposure was a result of intentional self-harm. Data from NPDS used in our analyses reflect the daily number of calls for exposures from any substance with a self-harm intent (fatal and non-fatal), normalized by dividing by the daily total of all exposures called into the system. The timeframe of this dataset was 2014-2017and constitutes a valuable signal capturing self-directed violence.

Datasets from Economics and Meteorology

There have been prior efforts to explore the relationship between suicides and economic indicators or meteorological patterns, such as daylight hours. For example, multi-year ecologic studies have identified a relationship between unemployment as well as economic recessions on suicide mortality rates.[3] Consequently, we also investigated whether such data can be used effectively in our estimation framework.

- **Economic datasets:** We first investigated multiple economic indicators available through Federal Reserve Economic Data (FRED; https://fred.stlouisfed.org/) and selected economic indicators based on three criteria: (i) the temporal granularity of the time-series dataset should be equal to or finer than monthly-level since more coarse-grained (e.g., quarterly or annual) time-series data would not be useful for estimating weekly suicide fatalities, (ii) the economic indicator should be available as raw data without transformation (e.g., seasonal adjustment), and (iii) the economic indicator should have a plausible theoretical relationship with suicide. eTable 1 lists the economic indicators meeting these criteria. All of the economic indicators studied are available monthly; hence, it was necessary to transform the datasets to estimate weekly suicide fatalities. Specifically, indicators released monthly were first used to calculate daily values by linearly interpolating (using state-of-the-art imputation techniques) between the consecutive two monthly values. We then extracted and used the value at the first day of each week (i.e., values on every Sunday) as the weekly indicator. A few economic indicators related to prices or wages (e.g., hourly earnings, home price index) are affected by inflation, which requires adjustment. Thus, we adjusted weekly earnings and home price index based on Consumer Price Index (CPI), which is also available from FRED (https://fred.stlouisfed.org/series/CPIAUCNS). The timeframe of this dataset was 2014-2017.
- **Daylight Hours:** The duration of daylight hours, calculated as the temporal difference between sunrise and sunset, has been considered as a predictor of the number of suicide fatalities[4] as both variables show similar seasonality; higher and lower in summer and winter, respectively. To test the number of daylight hours as a predictor, we collected the duration of daylight hours by using the times of sunrise and sunset from the application programming interface (API) described in eTable 1. As the U.S. encompasses a large geographic area, we retrieved the duration of daylight hours at the geographic center of the USA and used the averaged duration of daylight hours of each week as our predictor variable. The timeframe of this dataset was 2014-2017.

Datasets from Online Sources

Research over the past decade has situated social media platforms like Twitter to provide a reflection of social cohesion and mood of millions of individuals during the course of their daily lives.[4,5] Additionally, with the rising rates of social media use across different demographic groups,[6] research has shown that it is possible to gather meaningful cues about people's naturalistic behavior, affect, cognition, and sociality, unobtrusively and cheaply, and quickly, at a scale and granularity not possible before. Drawing on more relevant prior research in mental health and social media,[7–10] that reveals potential of data from social media platforms to inform risk estimation and prediction models (see Dredze[11] for an overview of the general role of social media in public health, and Chancellor and De Choudhury[12] specifically on the use of this data for mental health), in this work, we employed a variety of social media and online signals.

To gather suicide-relevant signals in social media or web services, we conducted keyword-based searching for 5 widely used social media platforms or web services[13] – Google Trends, YouTube Trends, Twitter, Tumblr, and Reddit. These platforms have been used in prior research to understand mental health as well as to assess suicidal risk.[7–10] They have also been found to be extensively used for health related information seeking and consumption,[14, 15] with over 60% of internet users reporting to go online to look for health information.[16] We also note that, individually, almost none of these platforms are perfectly representative of the U.S. general population.[17] However, we note that they target slightly different population segments. For instance, Twitter users' median age is 40 years with females and Blacks represented more frequently than the U.S. general population,[18] Tumblr is largely popular among teens

©2020 Choi D et al. *JAMA Network Open*.

and young adults,[19] while Reddit is widely used by urban males.[20] Therefore, a comprehensive approach like ours that integrates these data streams can likely counteract significant skewness in representativeness of specific subgroups.

To gather data from these 5 sites, keyword lists (see eTable 2) were built by drawing from prior literature[21,22] using each data source and followed by manual review and curation of assembled lists by domain (public health) experts (SS, KH). These keywords consisted of pro-suicide terms, suicide prevention terms, terms relating to mental health state and risk factors, as well as other terms expressing socio-economic disadvantage. Using guidance from Tran et al,[23] we ensured we excluded keywords that were about movie, band or song titles, words that are used in a lyrical, humorous, or flippant context, and opaque or rare phrases. We describe the collection methodology of each social media or web services below. The descriptive statistics of the online datasets are summarized in eTable 3. Since each service provides different types of datasets (e.g., tweets, trend scores, or posts) in different ways, we implemented automated crawlers for individual services and calculated time series data (e.g, number of posts or users) from the collected datasets at week granularity. To extract the distinct patterns of each service, we finally normalized the dataset based on their usage statistics publicly available on Statista (https://www.statista.com/markets/).

- **Google and YouTube Trends:** Keywords used for both Google and YouTube trends were informed by prior literature as noted above .[24–26] For each keyword, Google Trends[27] provides a time series of normalized scores ranging from 0-100 which indicates the popularity of a given word in search data for a particular week in time relative to all other weeks examined[1]. To collect the weekly trend scores of the 42 suicide-relevant keywords in Google, we used the *pytrends* python library to access data from Google Trends. We obtained scores for all of the keywords in three categories provided by Google: "All categories", "Health", and "Mental Health": see[28] for all the available categories. In a given category, scores for each of the individual keywords were then summed for each week, yield- ing a single final numerical score for each week for the category. After computing the weekly scores for these three categories, we evaluated each of these datasets at the first stage of the proposed machine learning framework using only the training and validation data. For Google Trends selected "All categories" as it outperforms the models from the other categories. We repeated this procedure using YouTube as a target service and selected the "Mental Health" category using the same procedure. The timeframe of this dataset was 2014-2017.

- **Reddit:** Although Reddit supports an open policy that allows collection of all posts, comments, and user data on the platform, the official API returns only 1,000 recent posts in order to prevent performance degradation. Given growing needs to access historical Reddit data, a Reddit user, Jason Baumgartner, a few years ago, launched *PushShift. PushShift* is a service which not only stores longitudinal historical data from 2008 till recent time, including posts, comments, and users on Reddit, but also provides APIs for systematic acquisition of this data. Drawing on prior mental health research on Reddit,[29] we identified 55 suicide-related message boards or communities, known as subreddits. Using the publicly available *PushShift* API, we calculated the total number of weekly posts made on these subreddits and normalized the data by dividing by the total number of posts across all subreddits on Reddit for each week. The timeframe of this dataset was 2014-2017.

- **Twitter:** Building on prior literature ,[30] we collected tweets for 38 suicide-relevant keywords. Since it is challenging to ascertain whether the total weekly volume of Twitter messages should be normalized and how, we first computed three types of input streams from the collected data: (i) weekly volume of Twitter messages, (ii) weekly number of unique users, and (iii) weekly number of unique users normalized by using estimates of the total Twitter user base.[31] We then examined these three streams in the first phase of the proposed machine learning pipeline using only the training and validation data. We selected the first stream – the total weekly volume of Twitter messages for subsequent analysis. The timeframe of this dataset was 2014-2017.

- **Tumblr:** To collect suicide-related posts from Tumblr, we used a list of 42 hashtags, drawn from prior research.[32] Using the official Tumblr API we collected all posts that contain at least one of these hashtags. We then calculated the weekly number of posts, normalizing the data by dividing each week's value by the estimated total number of weekly posts on Tumblr. This denominator for normalization was calculated by linear interpolation from annual estimates of posts on Tumblr.[33] The timeframe of this dataset was 2014-2017.

[1]We note the following limitation with the use of Google Trends and YouTube Trends: these services do not provide raw counts of search volume, instead provide normalized values. This is in contrast to the social media platforms considered in this research, where we can obtain crude posting volume across populations of interest. An artifact of this black box nature of the Google/YouTube Trends service is that trends requested for earlier years can be slightly asynchronous compared to later years. Moreover, trends obtained on different days may result in slightly varying results for the same week, because of the underlying normalization. To tackle these issues, we adopted the measures outlined by Tran et al.[23]

©2020 Choi D et al. *JAMA Network Open*.

Online Data Sources Not Considered We note that in this work we have not used data from some of the most popular social media platforms, such as Facebook; YouTube and Facebook are the most-widely used online platforms, and their user bases are broadly representative of the U.S. population as a whole.[13] Although Facebook data in recent years has been shown to be useful in various public health studies,[34, 35] this data is not publicly accessible at population-scale through Facebook's APIs. Further, we did not consider Instagram, Pinterest, Snapchat, LinkedIn, and WhatsApp that are the other popular social media sites in the U.S.,[13] some of which are particularly widely used among adolescents and young adults – a population at a greater risk of suicide.[36] This was because a) data from these platforms also could not be included due to unavailability of API-supported acquisition capabilities; or b) they were not deemed to be insightful for identifying suicidal risk or suicidal behaviors (particularly Pinterest and LinkedIn). Despite these limitations and logistical and practical bottlenecks, since we intend the outcomes of this research to be translatable for public health surveillance of suicide in the general population, focusing on platforms that allowed automated (programmatic) acquisition of public data or trends without necessitating individual informed consent was an important consideration. In addition, data on many of these social media platforms are audience-controlled;[37] however, the three social media platforms considered here – Twitter, Reddit, and Tumblr – are microblogging, broadcasting, or public- facing platforms, that enable candid self-disclosure on stigmatizing topics like mental health and suicide.[38] For these reasons, data from these three platforms were deemed more relevant for this research.

eMethods 2. Machine Learning Model Development

Time Series Forecasting Models: Considerations and Limitations. To design the framework for estimating suicide fatality trends, we first considered conventional time-series forecasting models including Poisson process-based forecasting,[39] AutoRegressive (AR) models and related variants such as the AutoRegressive Integrated Moving Average (ARIMA), Vector AutoRegressive Moving Average (VARMA), and Seasonal AutoRegressive Moving Average (SARIMA). All of these models are based on the methods of Box and Jenkins[40] and are widely used in time-series forecasting problems due to their ease of implementation,[41] and ability to account for seasonality and underlying patterns in the data.[42] Unfortunately, the AR-associated models are not ideal for our particular suicide fatality estimation task given the unique constraints of the data used to make the predictions. In particular, the gold-standard suicide fatality data (outcome data) is released only one time per year as noted in the main article; specifically, data for the preceding year is released approximately each December of the subsequent year. For example, suicide fatality data for each week of 2017 is released at the end of December 2018. That is, since there is more than an year's worth of lag between when historical data is available and when estimates are being made, the practical estimation framework should predict the weekly numbers of suicide fatalities based on the historical data from the year before the target year of estimation. In formal terms, AR-associated models require the actual values at the previous step t-1 to predict the values at the given step (t) by their mathematical definition. Due to these real-world limitations in the underlying suicide fatality data and its episodic release, we did not employ AR models in our estimation framework. Moreover, AR models are also not considered very robust to outliers and missing data[43] – aspects likely to be true when multiple disparate data sources are combined. In fact, certain time series forecasting methods, more generally, do not adequately account for structural similarities (e.g., autocorrelational pattern, temporal trends) in the explanatory time series (e.g., signals from our various data streams) and the dependent time series (i.e., suicide fatalities).[23]

Recent research has therefore advocated machine learning techniques such as LSTMs (long-term short-term memory) and RNNs (recurrent neural networks) as a way to tackle these challenges, especially when the explanatory data streams constitute multivariate time series.[44] Our proposed approach is guided by these recommendations.

Rationale Behind an Ensemble Approach. As noted in the main manuscript, there are significant limitations to using single data sources for a computational approach to estimating public health and other population-level outcomes. For instance, Mislove et al[45] have argued that Twitter data tends to skew towards young, urban, minority individuals, while Gayo-Avello[46] showed that age bias can affect attempts to predict socio-political outcomes from Twitter sentiment. Most relevantly, recent research by Ernala et al[47] showed that many existing research that has leveraged social media data solely as a proxy for mental health indicators suffers from issues of poor construct validity, offering limited clinical value. Further, there could be additional variations in social media usage along a number of dimensions: low resource or low-income areas may not have as much social media penetration. The challenges and risks of using a single data source are, however, not just limited to social media. Data sources such as health services may have different types of biases, such as those who do not reach out for emergency services for suicidal thoughts or behaviors may not being using Google to search for suicide related topics, or those who express suicidal thoughts on social media may not

©2020 Choi D et al. *JAMA Network Open*.

call the national suicide prevention helpline during a crisis. Essentially, a variety of gaps like these have been raised by Lazer et al,[48] such as ignoring the foundations of such measurement, reliability, and dependencies among various datasets, and most notably, "big data hubris," the often implicit assumption that big data are a substitute for, rather than a supplement to, "small data", like traditional data collection and analysis. Summarily, as boyd and Crawford rightly noted, "bigger data are not always better data".[49]

Based on this intuition and observations in the existing literature, we consider social media and other data sources in conjunction with other data sources through ensemble approach. The rationale behind this is that when we consider these different but complementary signals, we will overcome the limitations and mis/under-representation of populations in any one of them alone. With this consideration, we determined that our model should meet the following criteria: (a) maximally extract the explanatory signals from each dataset; (b) estimate suicide trends by combining such extracted signals in an intelligent and harmonic way, which learns how to give weights to individual data sources; and (c) be robust to spurious correlations, missingness and sparsity, and outliers. From this consideration, we propose a machine learning pipeline consisting of two stages: (i) intermediate prediction stage and (ii) ensemble stage.

Our approach to adopt an ensemble strategy, based on state-of-the-art advancements in artificial neural networks, extends recent recommendations on harnessing large-scale data and computational techniques in healthcare.[50, 51] We note that neural network models belong to a data-driven approach, where training depends on the available data with little prior rationalization regarding relationships between variables.[52] They also do not make any assumptions about the nature of the distributions of the underlying time series. As a result, these models are self-adaptive and considered a good alternative to time series forecasting models like ARIMA, where some of the explanatory data may be non-linear time series.[53] Further, our rationale to adopt a two-phase pipeline is as follows. The standard approach of ensembling is simple averaging that assigns equal weights to all component models, and therefore could be accomplished in a single step pipeline instead of two.[54] However, this simple averaging method may be sensitive to outlier values and unreliable for skewed distributions, especially given the diversity of the various streams considered in this research (ref. S2). By adopting a two-step approach and neural network models, we are able to find the optimal weights (or contributions of the estimates from each stream) by minimizing the sum of squared errors (SSEs).

Proposed Design. For the first phase, or the intermediate prediction stage, we focused on finding the best predictor of the weekly number of suicide fatalities for each given data source, by taking a training-validation approach. To this end, we built multiple machine learning models, each of which predicts the weekly number of suicide fatalities based on the time-series values from a single data source. In particular, a model (for a given data source) is trained based on the time-series data from a single source (i.e., Twitter) and the weekly numbers of suicide fatalities, and then finally outputs the intermediate results (a prediction of the weekly number of suicide fatalities in the given week of interest). The second phase involves combining these predictions via an ensemble approach[55] to arrive at a single estimate of weekly suicide fatalities that incorporates signals from all of the different data sources. Essentially, the neural network model in the ensemble (second) stage accepts and is trained by the intermediate results from all the models in the first stage. It then estimates the weekly numbers of suicide fatalities from all of the intermediate predictions made from each individual data source and model. The rationale of a machine learning-based ensemble approach is to let the ensemble model weight and combine all the intermediate values in an automatic and intelligent way.

We acknowledge here that by design, this model is not interpretable and does not include explainability of the underlying decision-making complexities of the algorithms used. In machine learning research, interpretability and explainability are desired,[56] especially in the domain of health. These features allow users of these models to take a peek into specific attributes, patterns, or representations of the explanatory variables (the various data streams) that may lead to good, bad, or unexpected outcomes, or to simply render the model results actionable in the real world. Unfortunately, such model interpretability often comes at a cost – in terms of performance – more interpretable and explainable models, such as linear regression models, tend to be less powerful in comparison to techniques like neural networks.[57] The goal in this work was to build the most accurate estimation model of suicide fatalities that is possible, which drove our decision to tradeoff interpretability over performance. Future work can consider posthoc techniques applied on the trained models to meet domain-experts' interpretability or explainability needs.[58]

Finally, by design, our machine learning pipeline also does not consider more sophisticated features derived from the data – recall, we simply derive frequency-based explanatory variables from the various data streams. Our rationale is twofold. First, frequency-based features are typical in prior suicide and other public health surveillance research,[11] and our approach aligns with these existing efforts. Second, while language data and data about the users of the various social media platforms have been harnessed as predictive signals in prior work,[7, 9, 59–61] because of the diversity of the streams considered and their mutual incompatibility of linguistic and social norms, these features were not

leveraged in this research. That said, future research can consider sophisticated techniques to harmonize these disparate linguistic and social behaviors in suicidal risk/outcome prediction (such as using convolutional neural networks or word embeddings[62] that learn high-dimensional representations in a latent space). This will be especially valuable if the goal is to examine the presence or absence of specific risk factors in social media/online data, or perhaps to even discover previously unknown correlates of suicidal risk and fatalities in specific subpopulations.

Development of the Pipeline. Considering that statistics on suicide fatalities are released annually (as also noted in the Introduction section of the main manuscript), we assume the situation that only the suicide fatalities through 2016 are accessible and the suicide fatalities in 2017 have not released yet – therefore 2017 in the year whose statistics have to be estimated. Accordingly, we set aside weekly suicide fatality data from 2017 as our held-out test data, 2016 fatalities data for validation, and data from 2015 and before (if available) for training the machine learning models corresponding to each data source. Note that the validation is a one-time procedure in order to select the optimal hyper-parameters, which can then be used for model testing in subsequent years (here, the year 2017).With this set up, predictive features for training, validation, and testing for each of the health services and online data sources were constructed using a sliding window approach,[63] illustrated in eFigure 1. For Lifeline, Poison, Google, YouTube, Reddit, Tumblr, and all of the economic and meteorological datasets, we used weekly data over a 2-year sliding window as predictors when training, validating, or testing models. For ED Visit and Twitter data, weekly data over a single year sliding window time period were used due to data availability. Additionally, we leveraged historical data of weekly suicide fatalities as an additional data source for our estimation task, both to augment the estimates given by the real-time signals as well as to develop a baseline model for comparison. To preserve the natural data generation reality of this data source – as noted in the main article as well as above, this data is released in an episodic fashion only once a year – we avoided using data from the current year while estimating fatalities in the next year.

In this same first phase of our machine learning pipeline, we trained and validated a number of leading machine learning models, beginning from simpler, more interpretable models, and progressing to more sophisticated opaque models, for identifying the best predictor of weekly suicide fatalities corresponding to each data source. These included, Linear Regression,[64] Lasso,[65] Ridge Regression,[66] ElasticNet,[67] Random Forest,[68] and Support Vector Machine[69] models. Choosing this wide range of machine learning models also ensured that our ensuing results of estimated suicide fatalities are not an artifact of apriori selection of specific model types, as well as to ensure that the results are robust across different classes of machine learning approaches. As mentioned in the main manuscript, following standard machine learning procedures, we used an exhaustive grid search procedure to tune model parameters for each machine learning model and each data source, such as Ridge/Lasso/ElasticNet regression penalties on weight magnitudes, hyperparameters of the Random Forest, and the appropriate kernel for the Support Vector Machine regressor. All the parameters investigated during grid search are indicated in eTable 4.The best model for each source was selected as the one with the best performance among all candidate models, as determined by the Pearson correlation coefficient between the actual and the estimated weekly number of suicide fatalities in the validation period for the same data source.

For the baseline models, as noted in the main manuscript, we trained and validated three models that used the historical weekly counts of suicide fatalities – a Linear Regression model; a more complex machine learning model (Support Vector Machine), chosen empirically to be appropriate given the nature of the data and to be consistent with prior literature;[70] and a traditional time series forecasting model, the Holt-Winters method.[71] This method has been employed in prior epidemiologic work on predicting a variety of public health outcomes, including suicide.[72] The Holt-Winters method is highly suitable in our case because the method models three aspects of a time series: a typical value (average), a slope (trend) over time, and a cyclical repeating pattern (seasonality), expressed as three types of exponential smoothing – all of which are applicable in suicide fatalities data.

In the second (ensemble) phase, we combined the model predictions of weekly suicide fatalities given by each single data source from the first phase in an automatic and harmonic way via a Neural Network (NN) model, specifically a Multilayer Perceptron Model (MLP)[73], which consists of multiple layers with the same number of hidden units and uses Relu as an activation function. MLP models are memory-less, and they use the feed forward neural network architecture, which applies a supervised learning technique called back propagation algorithm for training the neural network.[74] Note that the rationale of this approach is in line with prior literature that suggests the "Super Learner" model.[75] That is, the model learns *how to give weights to different models* to harmonically combine the multiple ma- chine learning models. Inspired this, we built a NN-based ensemble model and fed the intermediate values given by the best unit model for each data source to obtain weekly estimates of weekly suicide fatalities in 2017 (our held-out test data). Similar to the first stage, we chose the hyper-parameters of the MLP models from the

©2020 Choi D et al. *JAMA Network Open*.

ones listed in the eTable 4 via the grid search algorithm at the validation step. Note that we measured all the possible combinations among three categories of data sources – (i) the model from the history of suicide fatalities (ii) the models of social media platforms, and (iii) the models of health services, and found that the ensemble of combining all the data sources in three categories show the highest performance.

eMethods 3. Exploration of Alternative Data Sets

We explored the feasibility of the datasets from the sources not included in our framework by measuring the estimation performance of the individual models, described in eTable 5. Only the CPI-adjusted Home Price Index and the number of daylight hours showed somewhat satisfactory Pearson correlation coefficients when deployed in an actual prediction model. Thus, we excluded the remaining economic data sources and a single social media data source (Tumblr) from consideration in our machine learning pipeline.

To be thorough, we observed if combining the Economic (Home Price Index) and Meteorological (daylight hours) data with the Health Services and Online data would improve estimations of suicide fatalities, as shown in eTable 6. Compared to the Baseline + Health Services + Online model, as reported in the results of Table 2 in the main article, we see that adding Home Price Index (HPI), the number of daylight hours, or both to this model degrades its performance.

There are several hypotheses that can be offered to explain these observations. First, although economic data has been shown in several cross-sectional studies to be associated with suicide,[1] broad economic indicators are still proxies for risk factors associated with suicide, whereas, for example, the health services indicators used represent actual clinical encounters for suicide-related behavior, and are thus simply a predictor that is closer to the outcome being studied. Moreover, the economic data themselves are often lagged indicators of suicide, and although they bear a general relationship with suicide outcomes, they lack the temporal immediacy needed to be useful in an estimation task over a finer temporal granularity – a granularity that is paramount for public health surveillance. Thus, combining a weak predictor with a strong one does not aid model accuracy; as noted previously, the quality of data is a key consideration in machine learning approaches beyond quantity of data.[49] With regards to the number of daylight hours, as noted in the main article, while this indicator has a strong Pearson correlation with suicides due to both being highly seasonal, the number of daylight hours from year-to-year in the U.S. is largely constant. Thus, by its nature, this indicator cannot predict year-to-year changes in the number of suicides, or long-term temporal increases or decreases in suicide rates, and will always bias predictions toward no net change in suicide counts.

©2020 Choi D et al. *JAMA Network Open*.

eFigure 1. Training, Validation, and Test Sets of All of the Data Sources

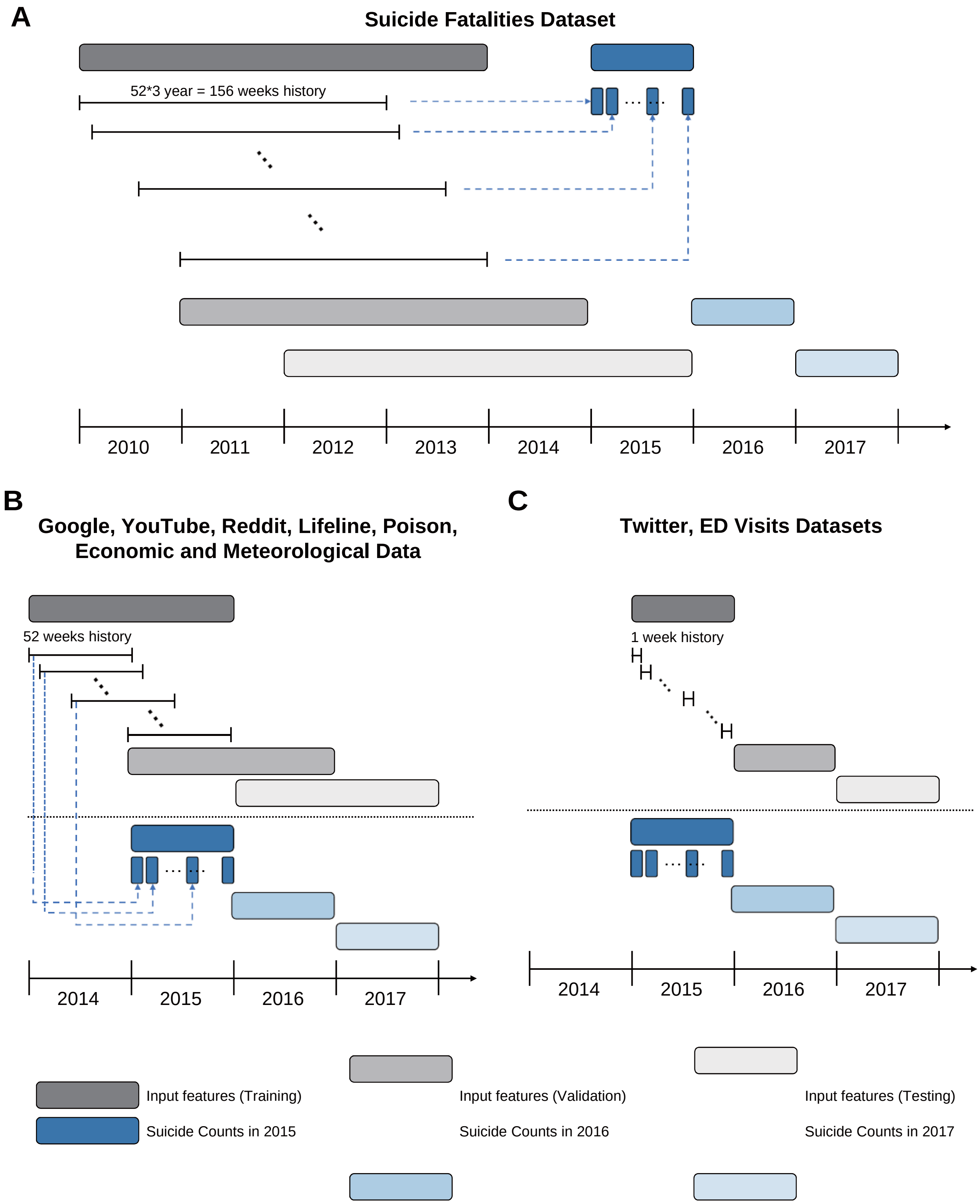

©2020 Choi D et al. *JAMA Network Open*.

eFigure 2. Weekly Number of Suicide Fatalities Estimated by the Different Ensemble Models

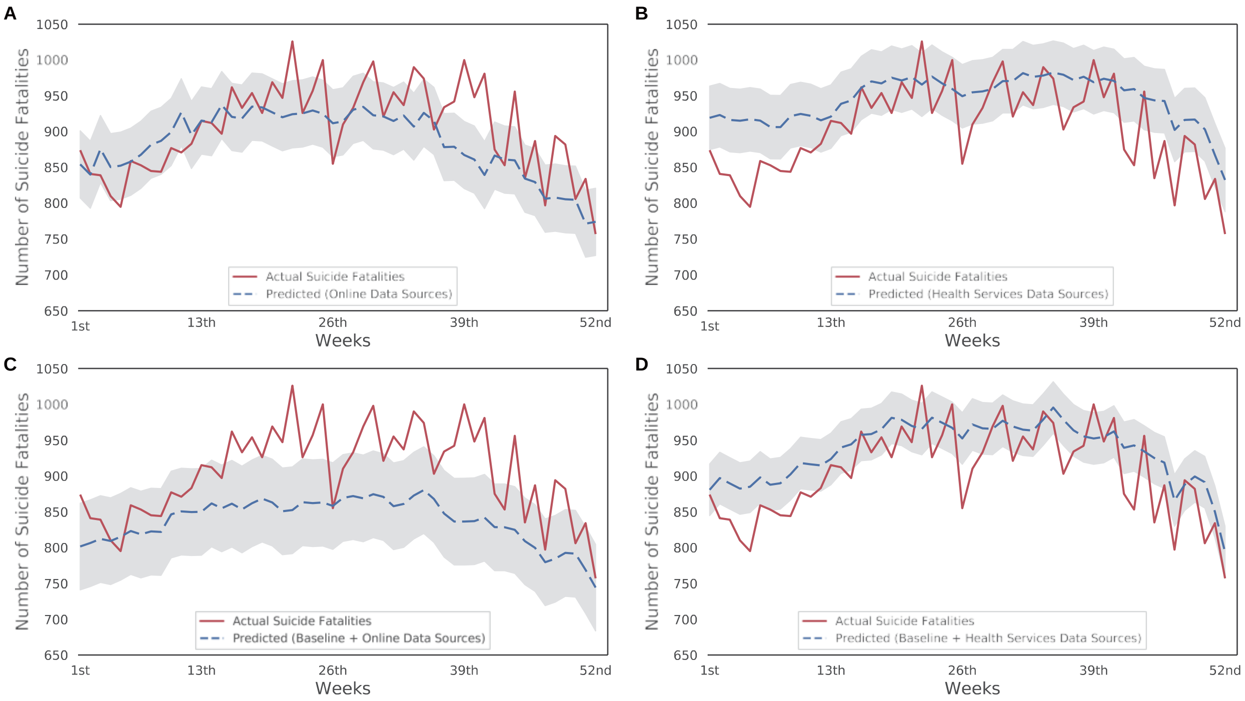

eFigure 2. Weekly Number of Suicide Fatalities Estimated by the Different Ensemble Models

eFigure 3. Time Series Values For Historical, Social Media, and Health Services Data

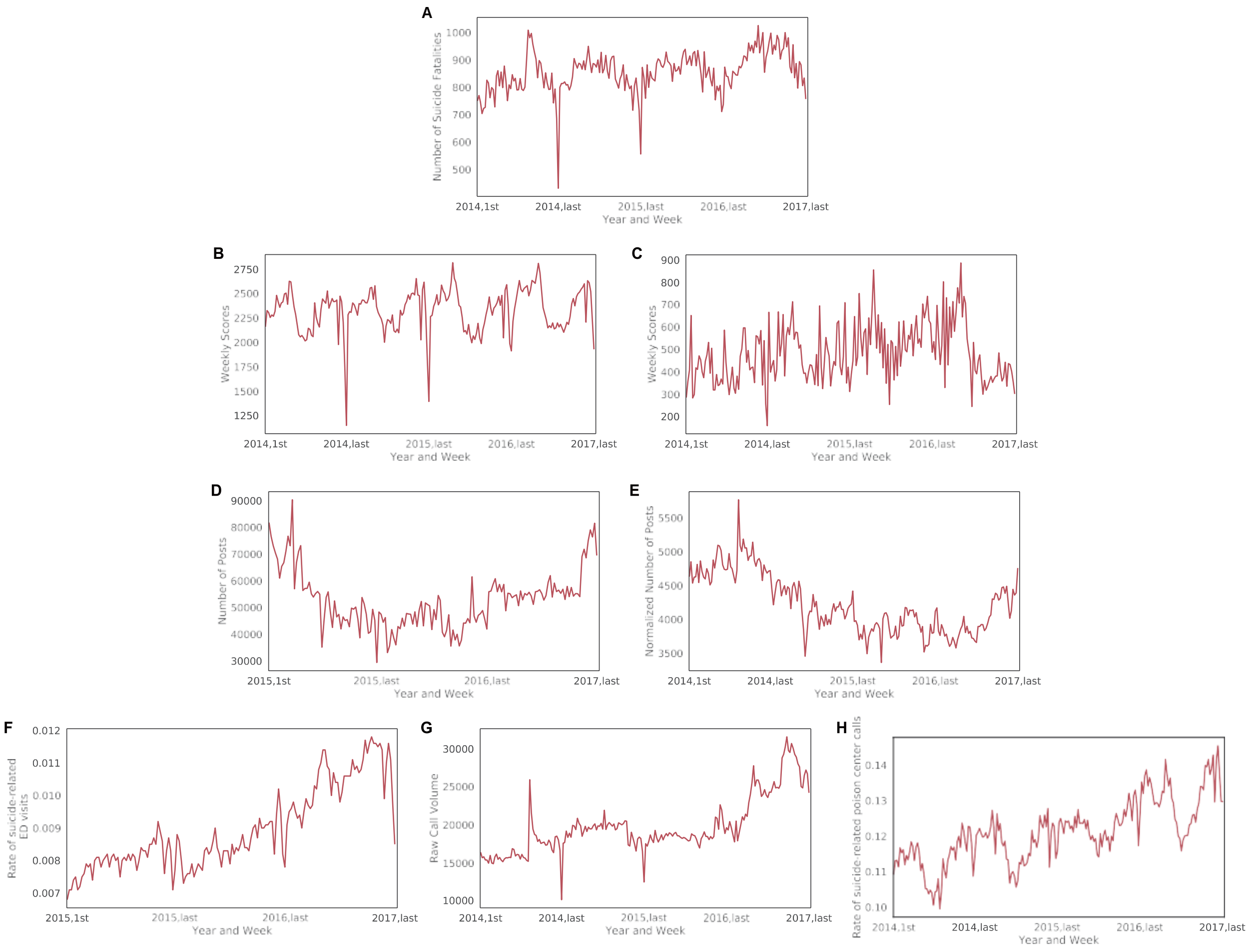

The time-series values of the history of suicide fatalities (row 1), social media platforms (row 2 and 3), and health services (row 4), which are included in the proposed model: (A) suicide fatalities, (B) Google (All categories), (C) YouTube (Mental Health category), (D) Reddit, (E) Twitter, (F) ED visits, (G) Lifeline, and (H) Poison datasets obtained by the methodology described in S1.

©2020 Choi D et al. *JAMA Network Open*.

eFigure 4. Time Series Values of Meteorology, Social Media Platform, and Economics Data

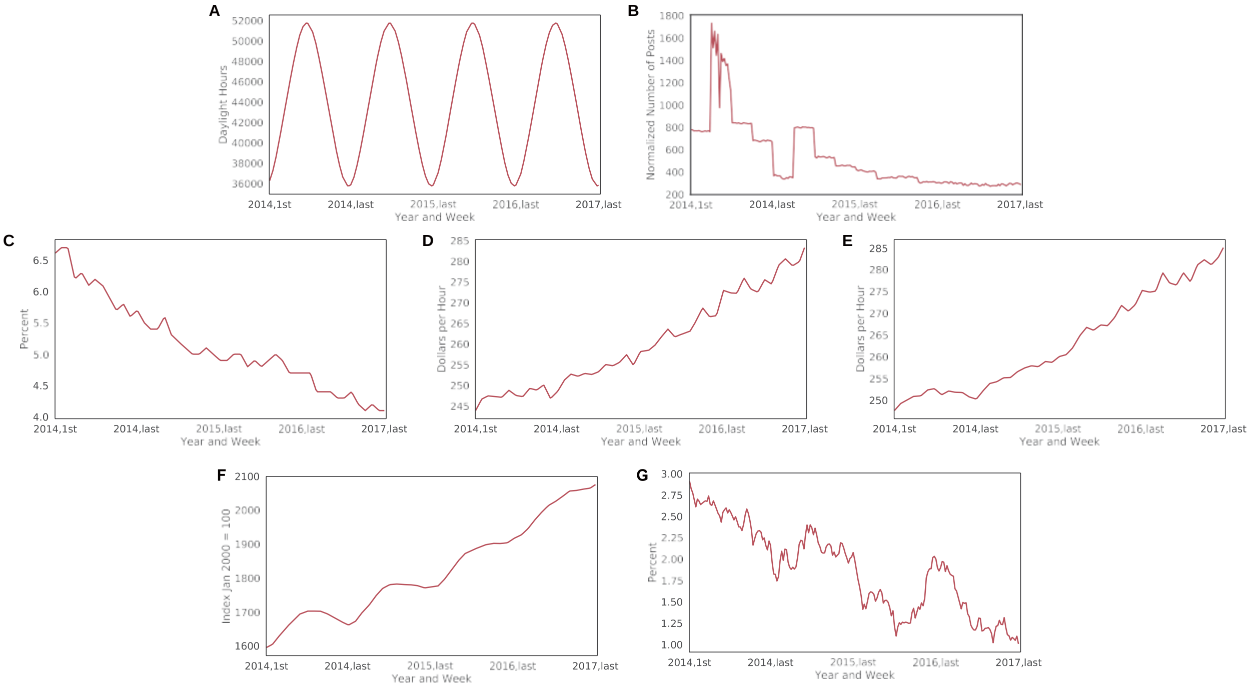

eFigure 4:The time-series values of the datasets from Meteorology, social media platform, and Economics, which are excluded in the proposed model: (A) daylight hours, (B) Tumblr, (C) average unemployment rate, (D) hourly earnings (all private), (E) hourly earnings (manufacturing), (F) housing, (G) 10 year-3 month treasury yield, obtained by the methodology described in S1. The values for Hourly Earnings and Housing Price Index (D-F) are adjusted for inflation by using the Consumer Price Index (CPI).

eTable 1. Socioeconomic-Behavioral Data Types and Their Associated Sources

Economic and Meteorological Data Types and Sources	Sources	Interpolation	CPI-adjusted
Avg. Unemployment Rate	https://fred.stlouisfed.org/series/UNRATE	Monthly to Weekly	No
Hourly Earnings (All)	https://fred.stlouisfed.org/series/CEU0500000003	Monthly to Weekly	Yes
Hourly Earnings (Manufacturing)	https://fred.stlouisfed.org/series/CEU3000000003	Monthly to Weekly	Yes
U.S. National Home Price Index (HPI)	https://fred.stlouisfed.org/series/CSUSHPINSA	Monthly to Weekly	Yes
10 Year-3 Month Treasury Yield	https://fred.stlouisfed.org/series/T10Y3M	Monthly to Weekly	No
Daylight hours	https://sunrise-sunset.org/api	Daily to Weekly	No

©2020 Choi D et al. *JAMA Network Open*.

eTable 2. Keyword Sets (or Subreddits) for Suicide-Related Data Collection for the 5 Online Data Sources

Data Source	Collection Criteria	
Google (YouTube)	Keywords	suicide, how to suicide, how to kill yourself, how to commit suicide, painless suicide, depression, depression and help, suicide and help, major depression, bipolar disorder, schizophrenia, anxiety disorder, stress, illicit drugs, alcohol, drunkenness, alcohol abstinence, insomnia, antidepressants, psychiatric service, therapy, marriage, divorce, abuse, domestic violence, relationship breakup, job, unemployment, social welfare, social benefits, religious belief, stock market, US Economy, lawsuit, asthma, allergy, pain, headache, cancer, chronic illness, I am suicidal, I am depressed
Twitter	Keywords	hang myself, stab myself, drug myself, ready to die, take my life, shoot myself, end this pain, ending it all, stop the pain, never wake up, sleep forever, poison myself, killing myself, to hurt myself, my death would, want to end it, cutting myself, die in my sleep, to live anymore, want to be gone, take it anymore, better off dead, tired of living, take my own life, not worth living, feeling hopeless, dont want to live, isnt worth living, dont want to exist, dont want to go on, want it to be over, my life isnt worth, put an end to this, nothing to live for, my life is pointless, dont want to wake up, not want to be alive, asleep and never wake
Reddit	Subreddits	r/abuse, r/adultsurvivors, r/afterthesilence, r/Anger, r/traumatoolbox r/bullying, r/CPTSD, r/domesticviolence, r/emotionalabuse, r/ptsd, r/PTSDCombat, r/rapecounseling, r/StopSelfHarm, r/survivorsofabuse, r/SurvivorsUnited, r/Agoraphobia, r/Anxiety, r/BipolarReddit, r/BipolarSOs, r/BPD, r/dpdr, r/psychoticreddit, r/MaladaptiveDreaming, r/Psychosis, r/PanicParty, r/schizophrenia, r/socialanxiety, r/calmhands, r/CompulsiveSkinPicking, r/OCD, r/Trichsters, r/7CupsofTea, r/BackOnYourFeet, r/Existential_crisis, r/getting_over_it, r/GriefSupport, r/helpmecope, r/hardshipmates, r/HereToHelp, r/itgetsbetter, r/LostALovedOne, r/offmychest, r/MMFB, r/Miscarriage, r/reasonstolive, r/SuicideBereavement, r/therapy, r/depression, r/depressed, r/ForeverAlone, r/GFD, r/lonely, r/mentalhealth, r/Radical_Mental_Health, r/SuicideWatch
Tumblr	Hashtags	anxiety, depression, mentalhealth, mentalillness, bipolar, bpd, ptsd, schizophrenia, panicattack, panic, anxietyattack, schizophrenic, schizo, socialanxiety, selfharm, mentaldisorder, paranoia, eatingdisorder, bingeeatingdisorder, anorexia, bulimia, anorexic, bulimic, depressed, blithe, suicidal, thinspo, suicide, cutting, selfhate, cutter, secret_society123, thighgap, thinspiration, killme, thinstagram, unwanted, depressing, stress, skinny, blades, blithe

eTable 3. Descriptive Statistics of Online Data Sets

Data Source	Period	Amount	Extracted Features
Google	2014-2017	-	Weekly summed scores of 42 suicide-relevant keywords in “All categories” on Web search
YouTube	2014-2017	-	Weekly summed scores of 42 suicide-relevant keywords in “Mental Health” category on YouTube search
Twitter	2015-2017	9,327,472 tweets from 5,565,341 users	Weekly numbers of tweets/retweets
Reddit	2014-2017	2,314,533 posts from 638,657 users	Weekly number of posts uploaded in 55 suicide-relevant subreddits, normalized by the number of all posts in Reddit
Tumblr	2014-2017	1,670,378 posts from 501,924 users	Weekly number of posts including at least one of 42 keywords, normalized by the number of all posts in Tumblr

©2020 Choi D et al. *JAMA Network Open*.

eTable 4. The List of the Parameters Explored During Grid Search at the First and Second Stages

Models	Hyperparameter Type	Investigated Values
ElasticNet	Alpha	[0.0, 0.1, 0.2, 0.3, 0.4, 0.5, 0.6, 0.7, 0.8, 0.9, 1.0]
	L1 Ratio	[0.1, 0.2, 0.3, 0.4, 0.5, 0.6, 0.7, 0.8, 0.9, 1.0]
Random Forest	Num. Estimators	[200, 500, 1000, 2000]
	Min. Sample Splits	[2, 5, 10]
	Min. Sample Leafs	[1, 2, 3]
Support Vector Machine	Kernel Func.	[Linear, Polynomial, RBF, Sigmoid]
	Gamma	[0.001, 0.01, 0.1, 1]
	C	[0.001, 0.01, 0.1, 1, 10, 100]
	Epsilon	[0.0001, 0.001, 0.01, 0.1]
Lasso	Alpha	[0.1, 0.2, 0.3, 0.4, 0.5, 0.6, 0.7, 0.8, 0.9, 1.0]
Ridge	Alpha	[0.1, 0.2, 0.3, 0.4, 0.5, 0.6, 0.7, 0.8, 0.9, 1.0]
	Solver	[Singular Value Decomposition, Cholesky, Regularized Least-Squares Routine, Conjugate Gradient Solver, Stochastic Average Gradient (Advanced Version)]
MLP (NN)	Num. Layers	[1, 2, 3, 4]
	Learning Rate	[0.0001, 0.001, 0.01, 0.1]

©2020 Choi D et al. *JAMA Network Open*.

eTable 5. Estimation Results From Individual Data Sources That Were Excluded in the Final Machine Learning Pipeline

Category	Source	Model	Parameters	Pearson Coeff.	RMSE	MAPE	SMAPE	Annual Est. Rate (Error %)
Meteorological Sources	Daylight Hours	Random Forest	#estimators:200, min. #samples of splits:10 min. #samples of leaf:2	0.699	76.011	6.877	3.611	13.46 (7.04%)
Economic Sources	Avg. Unemployment Rate	Ridge	alpha:0.1, solver: SVD	-0.36	389.675	37.22	24.324	9.69 (33.08%)
	Hourly Earnings (All)	Linear Regression		0.264	1059.802	114.529	90.794	-2.01 (113.88%)
	Hourly Earnings (Manufacturing)	Support Vector Regressor	C:1, ε:0.1, Gamma:1, Kernel:poly	0.204	18967.418	1726.414	94.249	-211.84 (1562.98%)
	Home Price Index (HPI)	Support Vector Regressor	C:0.1, ε:0.0001, Gamma:0.001, Kernel:poly	0.548	82.525	7.304	3.822	13.56 (6.35%)
	10 Year-3 Month Treasury Yield	Support Vector Regressor	C:1, ε:0.0001, Gamma:0.1, Kernel:sigmoid	-0.237	88.58	7.888	4.145	13.46 (7.04%)
Online Data	Tumblr	Support Vector Regressor	C:0.01, ε:0.0001, Gamma:1, Kernel:poly	-0.178	599.86	65.068	48.868	5.01 (65.40%)

©2020 Choi D et al. *JAMA Network Open*.

eTable 6. Performance of Ensemble Models Combining Home Price Index (HPI) and Number of Daylight Hours With Health Services and Online Data Sources

Ensemble Type	Pearson Coeff.	RMSE	MAPE	SMAPE	Annual Estimated Rate (Error %)
Baseline + Health Services + Online Data Sources + HPI	0.800	50.246	4.666	2.293	14.62 (0.97%)
Baseline + Health Services + Online Data Sources + Daylight Hours	0.806	54.568	5.136	2.477	15.03 (3.80%)
Baseline + Health Services + Online Data Sources + HPI + Daylight Hours	0.773	48.583	4.578	2.237	14.76 (1.93%)

©2020 Choi D et al. *JAMA Network Open*.

eTable 7. Performance of Models Built From Each Data Source at the Intermediate Stage (First Phase)

Category	Source	Model	Parameters	Pearson Coeff.	RMSE	MAPE	SMAPE	Annual Estimated Rate (Error %)
Historical Suicide Fatalities		Linear Regression		0.696	99.569	9.424	5.031	13.1 (9.53%)
		Support Vector Regressor	C:0.001, ε:0.0001, Gamma:1, Kernel:poly	0.761	79.79	7.485	3.941	13.35 (7.80%)
		Holt-Winter	Trend: None, Damped:None, Seasonal: Multiplicative, Seasonal Period: 52	0.759	84.087	8.029	4.238	13.27 (8.36%)
Health Services Data	Poison	Elastic Net	alpha: 1.0, L1 ratio:0.1	0.702	162.019	17.103	9.409	11.95 (17.47%)
	Lifeline	Elastic Net	alpha: 1.0, L1 ratio:0.1	0.491	83.871	7.256	3.419	15.27 (5.46%)
	ED Visits	Linear Regression		0.511	54.353	4.894	2.441	14.38 (0.69%)
Online Data	Google (All Categories)	Random Forest	#estimators:200, min. #samples of splits:2 min. #samples of leaf:4	0.721	85.04	7.846	4.135	13.28 (8.29%)
	YouTube (Mental Health)	Ridge	alpha: 1.0, Solver: Sparse CG	0.58	155.892	13.683	7.715	12.65 (12.64%)
	Reddit	Support Vector Regressor	C:100, ε:0.1, Gamma:0.01, Kernel:sigmoid	0.507	210.986	22.255	12.596	11.19 (22.72%)
	Twitter	Support Vector Regressor	C:100, ε:0.1, Gamma:1, Kernel:RBF	0.389	72.64	6.466	3.355	13.75 (5.04%)

©2020 Choi D et al. *JAMA Network Open*.

eReferences

[1] Ronald C Kessler, Guilherme Borges, and Ellen E Walters. Prevalence of and risk factors for lifetime suicide attempts in the national comorbidity survey. *Archives of general psychiatry*, 56(7):617–626, 1999.

[2] Marissa L Zwald, Kristin M Holland, Francis B Annor, Aaron Kite-Powell, Steven A Sumner, Daniel A Bowen, Alana M Vivolo-Kantor, Deborah M Stone, and Alex E Crosby. Syndromic surveillance of suicidal ideation and self-directed violence—united states, january 2017–december 2018. *Morbidity and Mortality Weekly Report*, 69(4):103, 2020.

[3] Aaron Reeves, David Stuckler, Martin McKee, David Gunnell, Shu-Sen Chang, and Sanjay Basu. Increase in state suicide rates in the usa during economic recession. *The Lancet*, 380(9856):1813–1814, 2012.

[4] Hong-Hee Won, Woojae Myung, Gil-Young Song, Won-Hee Lee, Jong-Won Kim, Bernard J Carroll, and Doh Kwan Kim. Predicting national suicide numbers with social media data. *PLoS One*, 8(4):e61809, 2013.

[5] J. Jashinsky, S. H. Burton, C. L. Hanson, J. West, C. Giraud-Carrier, M. D. Barnes, and T. Argyle. Tracking suicide risk factors through Twitter in the US. *Crisis*, 35(1):51–59, 2014.

[6] Shannon Greenwood, Andrew Perrin, and Maeve Duggan. Social media update 2016. *Pew Research Center*, 11(2):1–18, 2016.

[7] Munmun De Choudhury, Michael Gamon, Scott Counts, and Eric Horvitz. Predicting depression via social media. In *Seventh international AAAI conference on weblogs and social media*, 2013.

[8] Ladislav Kristoufek, Helen Susannah Moat, and Tobias Preis. Estimating suicide occurrence statistics using google trends. *EPJ data science*, 5(1):32, 2016.

[9] Munmun De Choudhury, Emre Kiciman, Mark Dredze, Glen Coppersmith, and Mrinal Kumar. Discovering shifts to suicidal ideation from mental health content in social media. In *Proceedings of the 2016 CHI conference on human factors in computing systems*, pages 2098–2110, 2016.

[10] Patricia A Cavazos-Rehg, Melissa J Krauss, Shaina J Sowles, Sarah Connolly, Carlos Rosas, Meghana Bharadwaj, Richard Grucza, and Laura J Bierut. An analysis of depression, self-harm, and suicidal ideation content on tumblr. *Crisis: The Journal of Crisis Intervention and Suicide Prevention*, 38(1):44, 2017.

[11] Mark Dredze. How social media will change public health. *IEEE Intelligent Systems*, 27(4):81–84, 2012.

[12] Stevie Chancellor and Munmun De Choudhury. Methods in predictive techniques for mental health status on social media: a critical review. *NPJ digital medicine*, 3(1):1–11, 2020.

[13] Jacob Poushter, Caldwell Bishop, and Hanyu Chwe. Social media use continues to rise in developing countries but plateaus across developed ones. *Pew Research Center*, 22:2–19, 2018.

[14] Wen-Ying Sylvia Chou, Yvonne M Hunt, Ellen B Beckjord, Richard P Moser, and Bradford W Hesse. Social media use in the united states: implications for health communication. *Journal of medical Internet research*, 11(4):e48, 2009.

[15] Munmun De Choudhury, Meredith Ringel Morris, and Ryen W White. Seeking and sharing health information online: comparing search engines and social media. In *Proceedings of the SIGCHI conference on human factors in computing systems*, pages 1365–1376, 2014.

[16] Rosemary Thackeray, Benjamin T Crookston, and Joshua H West. Correlates of health-related social media use among adults. *Journal of medical Internet research*, 15(1):e21, 2013.

[17] Jonathan Mellon and Christopher Prosser. Twitter and facebook are not representative of the general population: Political attitudes and demographics of british social media users. *Research & Politics*, 4(3):2053168017720008, 2017.

[18] Stefan Wojcik and Adam Hughes. Sizing up twitter users. *Washington, DC: Pew Research Center*, 2019.

©2020 Choi D et al. *JAMA Network Open*.

[19] Drew Desilver. facts about tumblr. *Pew Internet Report*, 5.

[20] William Sattelberg. The demographics of reddit: Who uses the site. *Tech Junkie*, 2019.

[21] John F Gunn III and David Lester. Using google searches on the internet to monitor suicidal behavior. *Journal of affective disorders*, 148(2-3):411–412, 2013.

[22] Benedikt Till and Thomas Niederkrotenthaler. Surfing for suicide methods and help: Content analysis of websites retrieved with search engines in austria and the united states. *The Journal of clinical psychiatry*, 2014.

[23] Ulrich S Tran, Rita Andel, Thomas Niederkrotenthaler, Benedikt Till, Vladeta Ajdacic-Gross, and Martin Voracek. Low validity of google trends for behavioral forecasting of national suicide rates. *PloS one*, 12(8):e0183149, 2017.

[24] Michael J McCarthy. Internet monitoring of suicide risk in the population. *Journal of affective disorders*, 122(3):277–279, 2010.

[25] V Chandler. Google and suicides: what can we learn about the use of internet to prevent suicides? *Public health*, 154:144–150, 2018.

[26] Albert C Yang, Shi-Jen Tsai, Norden E Huang, and Chung-Kang Peng. Association of internet search trends with suicide death in taipei city, taiwan, 2004–2009. *Journal of affective disorders*, 132(1-2):179–184, 2011.

[27] Google trends. Available at https://trends.google.com/trends/.

[28] Google trends categories. Available at `https://github.com/pat310/google-trends-api/wiki/Google-Trends-Categories`. Accessed January 30, 2020.

[29] Eva Sharma and Munmun De Choudhury. Mental health support and its relationship to linguistic accommodation in online communities. In *Proceedings of the 2018 CHI Conference on Human Factors in Computing Systems, CHI 2018, Montreal, QC, Canada, April 21-26, 2018*, page 641, 2018.

[30] Gualtiero B Colombo, Pete Burnap, Andrei Hodorog, and Jonathan Scourfield. Analysing the connectivity and communication of suicidal users on twitter. *Computer communications*, 73:291–300, 2016.

[31] Number of monthly active twitter users worldwide from 1st quarter 2010 to 1st quarter 2019 (in millions). Available at `https://www.statista.com/statistics/282087/number-of-monthly-active-twitter-users/`. Accessed September 4, 2019.

[32] Mrinal Kumar, Mark Dredze, Glen Coppersmith, and Munmun De Choudhury. Detecting changes in suicide content manifested in social media following celebrity suicides. In *HT*, pages 85–94. ACM, 2015.

[33] Cumulative total of tumblr posts between may 2011 and april 2019. Available at `https://www.statista.com/statistics/221565/total-cumulative-number-of-tumblr-posts/`. Accessed September 4, 2019.

[34] William R Hobbs, Moira Burke, Nicholas A Christakis, and James H Fowler. Online social integration is associated with reduced mortality risk. *Proceedings of the National Academy of Sciences*, 113(46):12980–12984, 2016.

[35] Steven A Sumner, Moira Burke, and Farshad Kooti. Adherence to suicide reporting guidelines by news shared on a social networking platform. *Proceedings of the National Academy of Sciences*, 2020.

[36] Holly Hedegaard, Sally C Curtin, and Margaret Warner. *Suicide rates in the United States continue to increase*. US Department of Health and Human Services, Centers for Disease Control and . . . , 2018.

[37] Liad Bareket-Bojmel, Simone Moran, and Golan Shahar. Strategic self-presentation on facebook: Personal motives and audience response to online behavior. *Computers in Human Behavior*, 55:788–795, 2016.

[38] Sindhu Kiranmai Ernala, Tristan Labetoulle, Fred Bane, Michael L Birnbaum, Asra F Rizvi, John M Kane, and Munmun De Choudhury. Characterizing audience engagement and assessing its impact on social media disclosures of mental illnesses. In *ICWSM*, 2018.

©2020 Choi D et al. *JAMA Network Open*.

[39] Tahmida Mahmud, Mahmudul Hasan, Anirban Chakraborty, and Amit K Roy-Chowdhury. A poisson process model for activity forecasting. In *2016 IEEE International Conference on Image Processing (ICIP)*, pages 3339–3343. IEEE, 2016.

[40] George EP Box, Gwilym M Jenkins, and Gregory C Reinsel. *Time series analysis: forecasting and control*, volume 734. John Wiley & Sons, 2011.

[41] David S Fink, Julian Santaella-Tenorio, and Katherine M Keyes. Increase in suicides the months after the death of robin williams in the us. *PLoS One*, 13(2), 2018.

[42] John C Robertson and Ellis W Tallman. Vector autoregressions: forecasting and reality. *Economic Review-Federal Reserve Bank of Atlanta*, 84(1):4, 1999.

[43] Wayne F Velicer and Suzanne M Colby. A comparison of missing-data procedures for arima time-series analysis. *Educational and Psychological Measurement*, 65(4):596–615, 2005.

[44] Nazanin Fouladgar and Kary Främling. A novel lstm for multivariate time series with massive missingness. *Sensors*, 20(10):2832, 2020.

[45] Alan Mislove, Sune Lehmann, Yong-Yeol Ahn, Jukka-Pekka Onnela, and J Niels Rosenquist. Understanding the demographics of twitter users. In *Fifth international AAAI conference on weblogs and social media*, 2011.

[46] Daniel Gayo-Avello, Panagiotis Takis Metaxas, and Eni Mustafaraj. Limits of electoral predictions using twitter. In *Fifth International AAAI Conference on Weblogs and Social Media*, 2011.

[47] Sindhu Kiranmai Ernala, Michael L Birnbaum, Kristin A Candan, Asra F Rizvi, William A Sterling, John M Kane, and Munmun De Choudhury. Methodological gaps in predicting mental health states from social media: Triangulating diagnostic signals. In *Proceedings of the 2019 CHI Conference on Human Factors in Computing Systems*, page 134. ACM, 2019.

[48] David Lazer, Ryan Kennedy, Gary King, and Alessandro Vespignani. The parable of google flu: traps in big data analysis. *Science*, 343(6176):1203–1205, 2014.

[49] Danah Boyd and Kate Crawford. Critical questions for big data: Provocations for a cultural, technological, and scholarly phenomenon. *Information, communication & society*, 15(5):662–679, 2012.

[50] Jacob M Montgomery, Florian M Hollenbach, and Michael D Ward. Calibrating ensemble forecasting models with sparse data in the social sciences. *International Journal of Forecasting*, 31(3):930–942, 2015.

[51] Shruti Kaushik, Abhinav Choudhury, Pankaj Kumar Sheron, Nataraj Dasgupta, Sayee Natarajan, Larry A Pickett, and Varun Dutt. Ai in healthcare: time-series forecasting using statistical, neural, and ensemble architectures. *Frontiers in Big Data*, 3:4, 2020.

[52] G Peter Zhang. Time series forecasting using a hybrid arima and neural network model. *Neurocomputing*, 50:159–175, 2003.

[53] Ayodele Ariyo Adebiyi, Aderemi Oluyinka Adewumi, and Charles Korede Ayo. Comparison of arima and artificial neural networks models for stock price prediction. *Journal of Applied Mathematics*, 2014, 2014.

[54] Ratnadip Adhikari and RK Agrawal. Performance evaluation of weights selection schemes for linear combination of multiple forecasts. *Artificial Intelligence Review*, 42(4):529–548, 2014.

[55] Cha Zhang and Yunqian Ma. *Ensemble machine learning: methods and applications*. Springer, 2012.

[56] Gajendra Jung Katuwal and Robert Chen. Machine learning model interpretability for precision medicine. *arXiv preprint arXiv:1610.09045*, 2016.

[57] Alfredo Vellido. The importance of interpretability and visualization in machine learning for applications in medicine and health care. *Neural Computing and Applications*, pages 1–15, 2019.

[58] Zachary C Lipton. The mythos of model interpretability. *Queue*, 16(3):31–57, 2018.

[59] Glen Coppersmith, Ryan Leary, Patrick Crutchley, and Alex Fine. Natural language processing of social media as screening for suicide risk. *Biomedical informatics insights*, 10:1178222618792860, 2018.

[60] Andrew G Reece and Christopher M Danforth. Instagram photos reveal predictive markers of depression. *EPJ Data Science*, 6(1):1–12, 2017.

[61] Johannes C Eichstaedt, Robert J Smith, Raina M Merchant, Lyle H Ungar, Patrick Crutchley, Daniel Preo¸tiuc-Pietro, David A Asch, and H Andrew Schwartz. Facebook language predicts depression in medical records. *Proceedings of the National Academy of Sciences*, 115(44):11203–11208, 2018.

[62] Jeffrey Pennington, Richard Socher, and Christopher Manning. Glove: Global vectors for word representation. In *Proceedings of the 2014 conference on empirical methods in natural language processing (EMNLP)*, pages 1532–1543, 2014.

[63] Chia-Shang James Chu. Time series segmentation: A sliding window approach. *Information Sciences*, 85(1):147 – 173, 1995.

[64] Xin Yan and Xiao Gang Su. *Linear Regression Analysis: Theory and Computing*. World Scientific Publishing Co., Inc., 2009.

[65] R. Tibshirani. Regression shrinkage and selection via the lasso. *Journal of the Royal Statistical Society (Series B)*, 58:267–288, 1996.

[66] Arthur E Hoerl and Robert W Kennard. Ridge regression: Biased estimation for nonorthogonal problems. *Technometrics*, 12(1):55–67, 1970.

[67] Hui Zou and Trevor Hastie. Regularization and variable selection via the elastic net. *Journal of the Royal Statistical Society, Series B*, 67:301–320, 2005.

[68] Andy Liaw and Matthew Wiener. Classification and Regression by randomForest. *R News*, 2(3):18–22, 2002.

[69] Alex J Smola and Bernhard Schölkopf. A tutorial on support vector regression. *Statistics and computing*, 14(3):199–222, 2004.

[70] Munmun De Choudhury, Scott Counts, and Eric Horvitz. Social media as a measurement tool of depression in populations. In *Proceedings of the 5th Annual ACM Web Science Conference*, pages 47–56. ACM, 2013.

[71] Roland Fried and Ann Cathrice George. *Exponential and Holt-Winters Smoothing*, pages 488–490. Springer Berlin Heidelberg, 2011.

[72] Jeffrey A Bridge, Joel B Greenhouse, Donna Ruch, Jack Stevens, John Ackerman, Arielle H Sheftall, Lisa M Horowitz, Kelly J Kelleher, and John V Campo. Association between the release of netflix's 13 reasons why and suicide rates in the united states: An interrupted time series analysis. *Journal of the American Academy of Child & Adolescent Psychiatry*, 2019.

[73] Richard O Duda, Peter E Hart, and David G Stork. *Pattern classification*. John Wiley & Sons, 2012.

[74] Timo Koskela, Mikko Lehtokangas, Jukka Saarinen, and Kimmo Kaski. Time series prediction with multilayer perceptron, fir and elman neural networks. In *Proceedings of the World Congress on Neural Networks*, pages 491–496. Citeseer, 1996.

[75] Mark Laan, Eric Polley, and Alan Hubbard. Super learner. *Statistical applications in genetics and molecular biology*, 6:Article25, 02 2007.

©2020 Choi D et al. *JAMA Network Open*.
